# Calcium sulfate-Cu^2+^ delivery system improves 3D-Printed calcium silicate artificial bone to repair large bone defects

**DOI:** 10.3389/fbioe.2023.1224557

**Published:** 2023-10-25

**Authors:** Shijie Gao, Jiawen Li, Qingjian Lei, Yan Chen, Huayi Huang, Feifei Yan, Lingfei Xiao, Tie Zhang, Linlong Wang, Renxiong Wei, Chao Hu

**Affiliations:** ^1^ Department of Spine Surgery and Musculoskeletal Tumor, Zhongnan Hospital of Wuhan University, Wuhan, Hubei, China; ^2^ Wuhan QISIDA Technology Development Co., Ltd., Wuhan, Hubei, China

**Keywords:** 3D printing, calcium silicate, calcium sulfate, Cu2+, bone repair

## Abstract

There are still limitations in artificial bone materials used in clinical practice, such as difficulty in repairing large bone defects, the mismatch between the degradation rate and tissue growth, difficulty in vascularization, an inability to address bone defects of various shapes, and risk of infection. To solve these problems, our group designed stereolithography (SLA) 3D-printed calcium silicate artificial bone improved by a calcium sulfate-Cu^2+^ delivery system. SLA technology endows the scaffold with a three-dimensional tunnel structure to induce cell migration to the center of the bone defect. The calcium sulfate-Cu^2+^ delivery system was introduced to enhance the osteogenic activity of calcium silicate. Rapid degradation of calcium sulfate (CS) induces early osteogenesis in the three-dimensional tunnel structure. Calcium silicate (CSi) which degrades slowly provides mechanical support and promotes bone formation in bone defect sites for a long time. The gradient degradation of these two components is perfectly matched to the rate of repair in large bone defects. On the other hand, the calcium sulfate delivery system can regularly release Cu^2+^ in the temporal and spatial dimensions, exerting a long-lasting antimicrobial effect and promoting vascular growth. This powerful 3D-printed calcium silicate artificial bone which has rich osteogenic activity is a promising material for treating large bone defects and has excellent potential for clinical application.

## 1 Introduction

In clinical treatment, successful bone healing is still not possible due to large bone loss, osteomyelitis, comorbidities, and aging (Holmes, 2017). The conventional clinical treatment for these incorrigible bone defects is bone grafting (Dalisson et al., 2021). In recent years, the large number of nonhealing bone cases have made bone the second most crucial tissue for transplantation after blood (Holmes, 2017; Lobb et al., 2019). The enormous demand for bone grafts has led to increased development of bionic bone materials. Stereolithography (SLA) 3D printing technology shows major advantages over numerous other bioprosthesis manufacturing processes for the preparation of structurally complex bionic bone materials for clinical cases (Ma et al., 2018). This technology can accurately design large scaffold structures and rapidly create personalized bone defect-filling models for patients (Quan et al., 2020; Cheng et al., 2021). We expected that blood and nutrients can be transported to the depths of large bone defects, and new bone can quickly grow into the center of large bone defects. This method will be a solution to the problem of complex bone repair.

Recent extensive research has found that calcium silicate has excellent properties in bone repair (Srinath et al., 2020). Silicon is strongly associated with bone density, bone mechanics, and possibly estrogenic status (Götz et al., 2019). Calcium silicate (CSi) can appropriately increase the silicon concentration and consistently induce bone regeneration and angiogenesis with degradation (Lu et al., 2022). These biological effects may promote repair at the bone defect site. For example, Chen et al. manufactured a multihole Si-CaP scaffold by digital light processing printing technology. The Si-CaP scaffold has a regular structure and excellent mechanical properties. This scaffold also promoted osteogenic factor expression and calcium deposition (Chen D. et al., 2022). This proves the excellent osteogenic effect of 3D-printed calcium silicate scaffolds.

The channel structure established by 3D printing technology has been studied in the field of bone repair. For example, the hollow tube structure has been proven to significantly promote the rapid infiltration of stem cells, cytokines and blood vessels to enhance tissue regeneration (Zhang et al., 2017). Zhang et al. established a 3D-printed Haversian structure that is conducive to early bone formation (Zhang et al., 2020). Based on these studies, we designed a three-dimensional tunnel structure. This horizontal and vertical traffic network can provide a pathway for cells migrating from all directions to the center of bone defects. However, the scaffold with hollow channels alone cannot quickly repair the empty area in the center of large bone defects because it is difficult for cells to traverse the distance of large defects. To solve the problem of the internal cavity of large bone defects, we introduced calcium sulfate (CS) into the three-dimensional tunnel structure to further enhance the osteogenic activity of the calcium silicate artificial bone. The rapid degradation and complete absorption of calcium sulfate contributes to the induction of osteogenesis by enhancing the expression of osteogenic cytokines (Chen H. et al., 2022; Chopra et al., 2023). Calcium sulfate also possesses unique osteoconductive properties that restore the morphological contours of bone (Echave et al., 2022). We expect that this factor will contribute to the gradual formation of native blood vessels and new bone tissue in the center of the scaffold. Zhang et al. directly added calcium sulfate into the calcium silicate printing stock, but the calcium sulfate decomposed under the high temperature of calcination and lost its biological activity (Zhang et al., 2021). We improved this scheme by filling calcium sulfate into the channels of the three-dimensional tunnel structure, ensuring rich biological functions.

We also took advantage of calcium sulfate degradation to construct a delivery system. The delivery system can slowly release drugs, further enhancing the biological activity of calcium silicate artificial bone. Postoperative infection is a key challenge that can lead to bone repair failure. Cu^2+^ is a well-known broad-spectrum antimicrobial material and has been shown to be effective against Gram-positive bacteria, Gram-negative bacteria, and fungi (Mitra et al., 2020). The addition of Cu^2+^ can effectively prevent infection problems during bone repair processes. Cu^2+^ also contributes to cell adhesion, osteogenic differentiation of mesenchymal stem cells, and production of vascular endothelial growth factor (VEGF) (Lin et al., 2016). These factors are all necessary for successful tissue regeneration. Therefore, we added Cu^2+^ to calcium sulfate to verify the performance of the delivery system.

**FIGURE 1:**
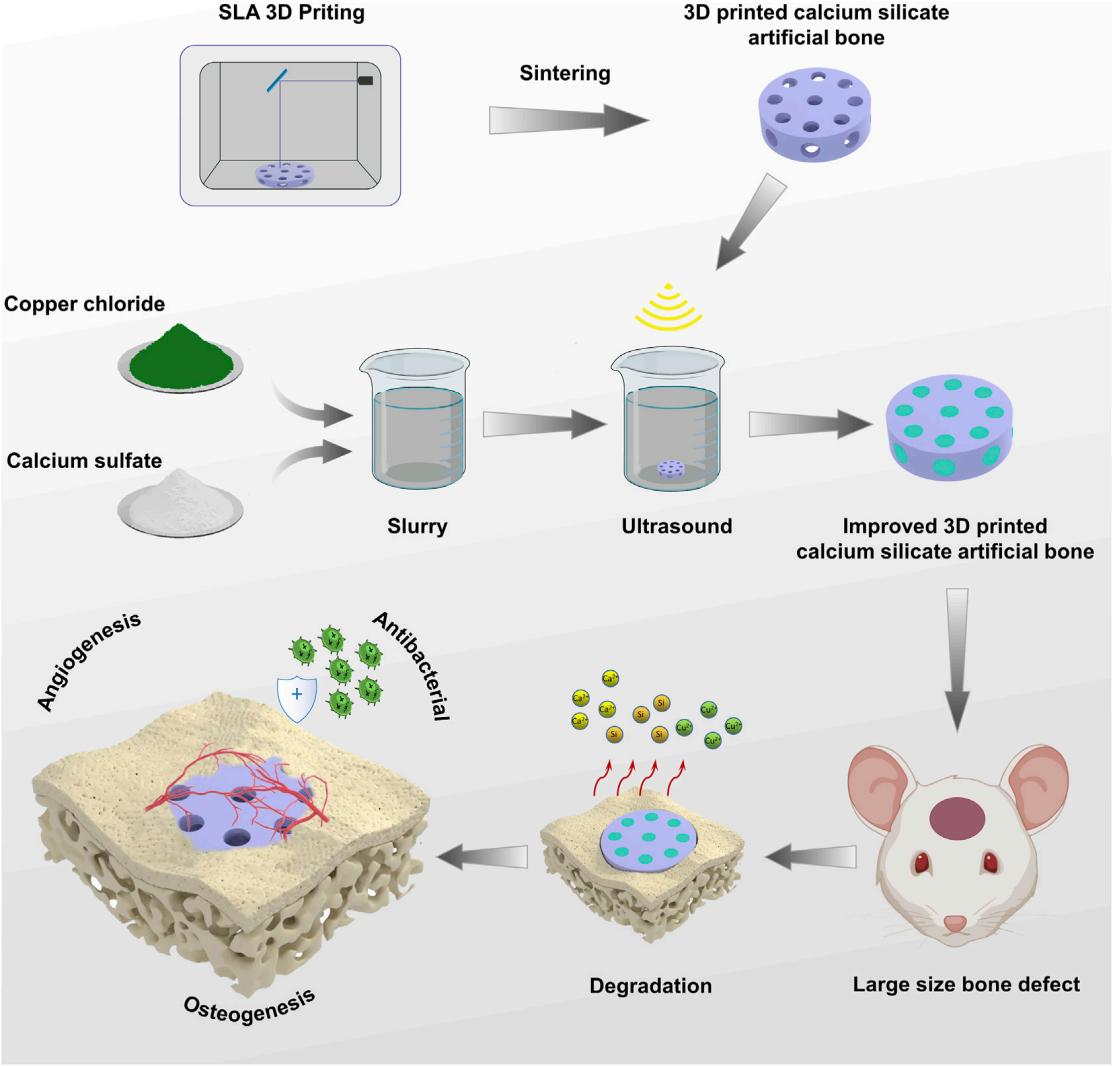
Schematic Illustrations: The preparation process of 3D-printed calcium silicate artificial bone improved by calcium sulfate-Cu^2+^ delivery system and its role in the bone defect area.

In summary, we printed a calcium silicate artificial bone with a precise three-dimensional tunnel structure by SLA technology and fitted the calcium sulfate-Cu^2+^ delivery system into the channel to create a sandwich structure. Figure 1 outlines the synthetic route of 3D-printed calcium silicate artificial bone improved by the calcium sulfate-Cu^2+^ delivery system and its effect in the bone defect area. To systematically test our hypothesis that improved calcium silicate artificial bone could promote uniform osteogenesis at the center of large bone defects, we set pure calcium silicate-based 3D-printed scaffolds as a control group and investigated the physical and mechanical properties of the novel 3D-printed calcium silicate artificial bone improved by the calcium sulfate-Cu^2+^ delivery system. Subsequently, we comprehensively examined the *in vitro* biocompatibility, osteogenesis, angiogenesis, and antibacterial properties of different scaffold materials. Finally, we chose a large cranial defect model of 7 mm in diameter to assess the *in vivo* bone repair capabilities of 3D-printed calcium silicate artificial bone.

## 2 Materials and methods

### 2.1 Materials

Copper (II) chloride (99%) was purchased from Sigma-Aldrich. Calcium sulfate hemihydrate (CSH) was synthesized in the laboratory. First, magnesium sulfate heptahydrate and sodium citrate were added to pure water and stirred evenly. Then alcium sulfate powder dihydrate was added to the solution and stirred for 10 min. The obtained solution was placed in the reactor (130°C, 0.19 MPa) for 5 h. After the reaction, the supernatant was decanted and dried at 100°C for 4 h to obtain calcium sulfate hemihydrate. Finally, it was crushed and sieved to obtain the powder.

### 2.2 Preparation of the scaffolds

We use Magics software to model and slice the desired structure. After mixing calcium silicate ceramic slurry with photosensitive resin, the scaffold was printed by a CeramBuilder 100pro (iLaser, China). The operating parameters were as follows: the viscosity of 4 Pa s, a laser scanning speed of 2,500 mm/s, a slice thickness of 40 μm, and single layer curing depth of 120 μm. Subsequently, the printed blank was degreased and sintered in a muffle furnace to obtain the CSi group. The sintering conditions of the samples were as follows: 30°C–300°C with a heating rate of 2°C/min and 2 h of holding time; 300°C–600°C with a heating rate of 2°C/min and 2 h of holding time; 600°C–1,200°C with a heating rate of 5°C/min and 2 h of holding time. Then the samples were cooled naturally at room temperature. Copper chloride powder (0.5 wt%, CuCl_2_) and CSH powder (99.5 wt%) were mixed well with water at a ratio of 2 g/mL to form a slurry. The CSi group was immersed in the slurry and ultrasonically shaken for 5 min to assist the filling of the slurry into the channels. Then the scaffold was removed from the mud and cured at 37°C for 24 h. Finally, the scaffold was dried to constant weight to obtain the 3D-printed calcium silicate loaded with calcium sulfate and Cu^2+^ (CSi/CS/0.5Cu group) at 80°C. The CSi/CS/0.1Cu was prepared with 0.1 wt% CuCl_2_ and the CSi/CS/1Cu was prepared with 1 wt% CuCl_2_. The CSi/CS was prepared by mixing the CSi and calcium sulfate slurry without copper chloride powder according to the above method.

### 2.3 Scanning electron microscopy (SEM) and X-ray diffraction (XRD)

The surface morphologies of the scaffolds were characterized using a field emission scanning electron microscope (Zeiss SIGMA, England). Before scanning, each scaffold was gold sprayed. An X-ray diffractomer (XPert Pro, Netherlands) was used to scan the crystal structure of the powder of the scaffolds.

### 2.4 Mechanical strength evaluation

The compression testing machine (CMT6503, SANS Testing Machines, Shenzhen, China) with a 10 kN load cell was used to perform mechanical tests on the 3D-printed calcium silicate artificial bone. The scaffold was fabricated the cylinder (diameter, 7 mm; height, 10 mm) and was compressed in the vertical direction at a crosshead speed of 1 mm/min in the compression test. The maximum load at failure was recorded. The formula P = F/S was used to calculate the compression strength P (MPa), where F was the load at failure (N), S was the cross-sectional area of the sample (mm^2^).

### 2.5 *In vitro* degradation of scaffolds

Phosphate buffered saline (PBS) at pH 7.4 was chosen to test the *in vitro* degradation of the scaffolds. Scaffolds were immersed in 37°C PBS at a 0.05 g/mL concentration ratio and then placed in a shaker at 72 rpm. The scaffolds were removed from PBS after degradation for 1, 7, 14, 21, and 28 days. Then the scaffolds were dried to constant weight in a drying oven at 100°C and the weight was measured. The weight loss was calculated by formula shown below: Weight loss = 100%×(W0−W1)/W0. W0 is the weight before immersion and W1 is the weight after every immersion.

### 2.6 Ion release and change in pH by the scaffold in pure water

The ion release test of the scaffold was evaluated by inductively coupled plasma-atomic emission spectrometry ((ICP‒AES, IRIS Intrepid II XSP, Thermo Elemental). Each scaffold was immersed in 10 mL ultrapure water at a concentration ratio of 0.05 g/mL at 37°C. Only 1 mL of liquid was taken for testing each time, and then 1 mL of ultrapure water was added to restore the experimental conditions. The concentrations of chemical elements (copper, calcium, silicon) in the collected liquids (Days 1, 7, 14, 21, and 28) were measured by ICP‒AES. The pH values at Days 1, 7, 14, 21, and 28 were measured by a pH meter (FE28, Mettler Toledo).

### 2.7 *In vitro* mineralization

The scaffolds were immersed in simulated body fluid (SBF) and shaken in a water bath at 37°C for 7 days. Then, the scaffolds were removed from the SBF and rinsed with deionized water. After drying in a 60°C oven for 24 h, the surface of the scaffolds was sprayed with gold. The surface morphologies of the scaffolds were characterized using SEM and energy dispersive spectroscopy (EDS, Oxford UltimMax 40, England).

### 2.8 Cell culture

Bone marrow mesenchymal stem cells (BMSCs) were isolated from the femurs and tibias of Sprague–Dawley rats (4 weeks old). Both ends of the bone were cut off and the marrow cavity was washed with culture media to obtain cells. The cells were cultured in alpha minimum essential media (alpha MEM) containing 10% (V/V) fetal bovine serum (FBS, Gibco, U.S.A.) and 1% (V/V) penicillin‒streptomycin. Human umbilical vein endothelial cells (HUVECs, ATCC CRL-1730) were cultured in endothelial cell media with 5% (V/V) FBS and 1% (V/V) penicillin‒streptomycin.

of both BMSCs and HUVECs were made by immersing the material in the media without FBS at 37°C for 1 d. After the soaked media was filtered with a 0.22 μm bacteria filter, FBS and penicillin‒streptomycin were added to the media. The cells of the three experimental groups (CSi group, CSi/CS group, CSi/CS/0.5Cu group) were cultured in their respective conditioned media. The control group represented BMSCs and HUVECs cultured in conventional media.

### 2.9 Biocompatibility

The *in vitro* cytotoxicity of the biological scaffolds was determined by MTT assays. The density of the cells in 96-well plates was adjusted to 2000 cells per well. Cells were cultured with different conditioned media for 1, 3, and 7 days. After that, 10 μL of MTT solution (0.5% MTT) was added to each well. Dimethyl sulfoxide was added to each well after 4 h, and the crystals were fully dissolved at low speed for 10 min on a shaking table. The absorbance values were measured at 490 nm using a plate reader (Multiscanfc, Thermo Scientific). Cell viability was the ratio of the OD value.

Furthermore, BMSCs were seeded on the surface of each scaffold to evaluate the effect of the scaffolds. The scaffolds were wetted in alpha MEM for 24 h in advance, and then 100 μL of cell suspension (1 × 10^7^ cells/mL) was evenly dropped on the surface of the scaffold. After incubation for 1 h, the culture media was replenished. The cells were cultured with scaffolds for 3 days. Then the cells were stained with phalloidin (Sigma-Aldrich) or calcein AM (Sigma-Aldrich). The scaffolds were viewed with a confocal laser scanning microscope (LSM).

### 2.10 Alkaline phosphatase staining and alizarin red staining

For evaluation of the effect of the scaffolds on the osteogenic differentiation of BMSCs, alizarin red (Cyagen) and BCIP/NBT alkaline phosphatase (ALP) color development kits (Beyotime) were used to stain cells. A total of 20,000 cells per well were seeded in 24-well plates with conditioned media. ALP was stained on the seventh day to reflect early osteogenesis. Calcium nodules were stained with alizarin red on the 14th day to reflect late osteogenesis.

### 2.11 Real-time quantitative polymerase chain reaction (RT-qPCR)

RT-qPCR evaluated the expression of genes. Cells were seeded in 6 well cell cultrue cluster by conditioned media for 3 days and 7 days. The total RNA of cells was extracted by Trizol reagent. Reverse transcrip-tion of mRNA was performed using a HiScript III RT SuperMix reverse transcription kit (Vazyme). RT-qPCR was performed using a 7,500 Real-Time PCR system (Applied Biosystems, USA) with ChamQ SYBR qPCR Master Mix (Vazyme). Glyceraldehyde-3-phosphate dehydrogenase (Gapdh) was used to normalize the DNA content of the samples. The delta-delta CT method was used to analyze the quantitative polymerase chain reaction results.

### 2.12 Western blot analysis

For Western blot analysis, 40 μg protein was loaded into each sample hole. The sample was separated by SDS-PAGE with 10% dissolution gel, and transferred to PVDF membranes. After sealing with 5% fat free milk for 1.5 h, the membranes were probed with antibodies at 4°C overnight. On the second day, after washing the PVDF membrane, the antibody conjugated with HRP was incubated at room temperature for 1 h. Proteins were observed by chemiluminescence method.

### 2.13 Tube formation experiment

A tube formation experiment was uesd to evaluate the ability of the scaffolds to promote angiogenesis *in vitro*. HUVECs (1 × 10^5^) were seeded on growth factor-reduced Matrigel (200 μL/well, Corning, US) in 24-well plates. Then the cells were cultured in an incubator for 12 h. HUVECs were labeled with calcein. The tube morphology was observed by fluorescence microscopy. ImageJ software was used to determine the mean of the tube formation parameter.

Angiography was used to observe the distribution of blood vessels in rats. The thoracic cavity was opened after the rats were euthanized. Saline was injected from the heart to replace the blood in the rat, and Microfil (Flowtech) was injected to replace saline. Subsequently, the rats were refrigerated at 4°C. The next day, the skull samples were taken, and the vessels on the skull surface were photographed.

### 2.14 Antibacterial experiments

Disc diffusion assays were used to evaluate the antibacterial properties of the scaffolds. The scaffolds of each group were immersed in LB broth media at 200 mg/mL for 1 d. Then the sterile disc was immersed in the conditioned LB broth media and dried. *Staphylococcus aureus* was cultured in LB broth media with shaking at 120 rpm at 37°C for 16 h. The bacterial liquid was diluted to 1 × 10^6^ CFU/mL with PBS. Then the diluted bacterial liquid (50 μL) was inoculated on the blood plate with a triangular rod, and sterile discs were pasted on the blood plates. Finally, the blood plates were placed an incubator for 24 h.

### 2.15 Animal model and scaffold implantation

We prepared Sprague‒Dawley rats (8 weeks old) as a large bone defect model. Rats were anesthetized by intraperitoneal injection of 2.5% sodium pentobarbital (40 mg/kg). The skin tissue was incised to reach the skull surface, and a 7 mm diameter circular defect was drilled in the middle of the skull with a motorized bone drill. Then the defective area was filled with scaffold and the cut was closed. After 6 and 12 weeks, the skulls were collected and immersed in 4% paraformaldehyde for fixation.

### 2.16 Micro-CT evaluation

To evaluate the repair of the bone defect model in animal experiments, we performed microcomputed tomography (micro-CT) scans with bone tissue at the 6th and 12th weeks to quantitatively evaluate bone healing. The samples were placed on the scanning platform and tested by micro-CT system (SkyScan 1,276 system, Bruker, Germany). After each sample was scanned, 3D reconstruction was performed using the Sky Scan program. Numerical analyses were then performed to calculate the bone volume percentage (BV/TV).

### 2.17 Histological analysis

Skull samples were immersed in ethylenediaminetetraacetic acid to decalcify for 2 weeks. Then the skull was embedded in paraffin. The tissues in the paraffin were cut to 5 µm by a slicer and pasted on the slides. The slides were stained with hematoxylin and eosin (H&E), and Masson trichrome to observe new bone production and neovascularization.

### 2.18 Statistical analysis

All experiments were repeated three times in each group. The data were all analyzed by GraphPad Prism 7.02. And the data were calculated as the mean ± standard deviation (SD) and analyzed by one-way analysis of variance (ANOVA) with Tukey’s test. Statistical significance was attained with a greater than 95% confidence level (*, *p* < 0.05; **, *p* < 0.01; ***, *p* < 0.001; ****, *p* < 0.0001).

## 3 Results and discussion

### 3.1 Structure and composition of the scaffolds

**FIGURE 2:**
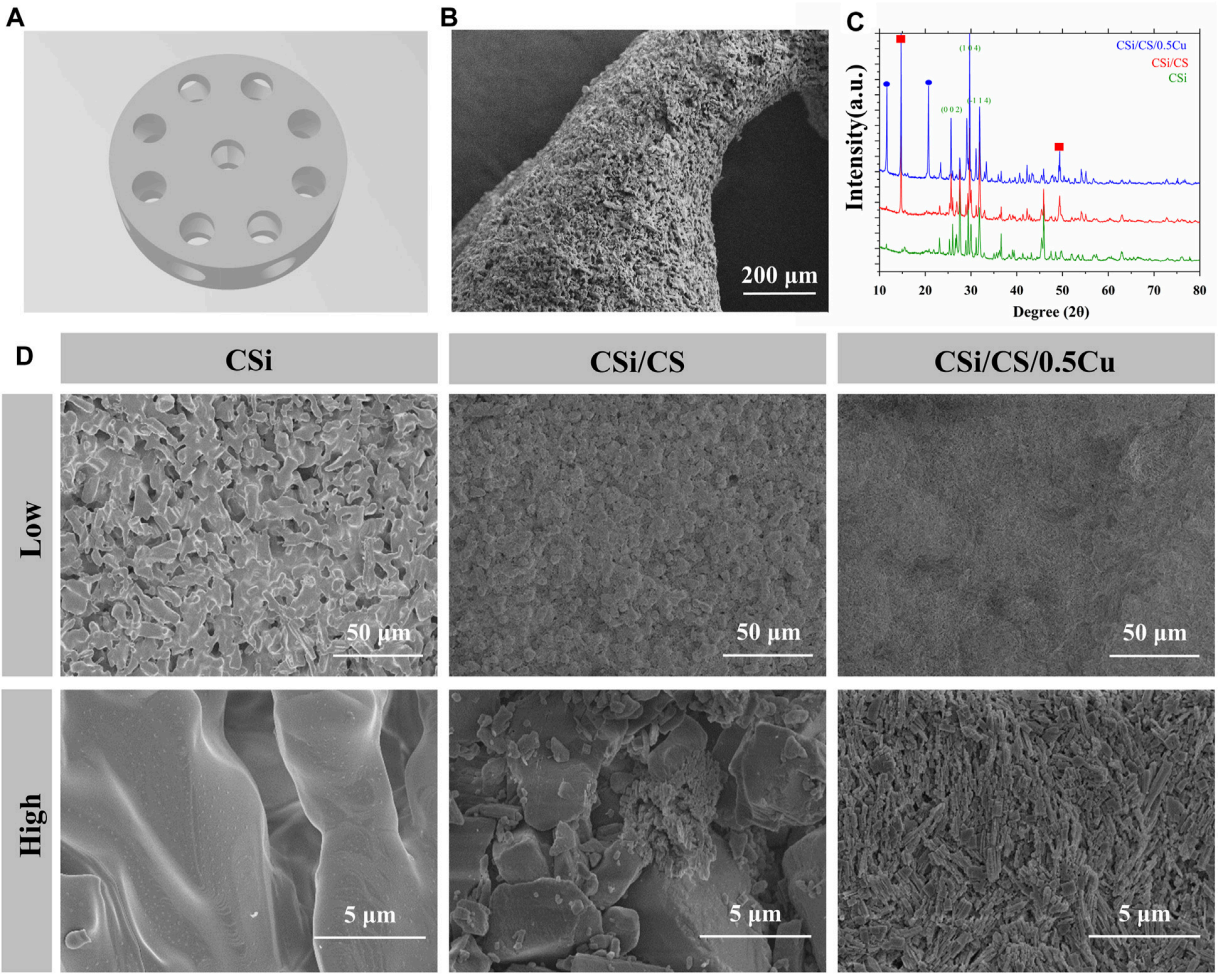
Structure and composition of the scaffolds. **(A)** 3D model of the CSi scaffold. **(B)** General view of the CSi scaffold under SEM. **(C)** XRD spectrum of the 3D printed scaffolds. **(D)** Representative SEM images of various groups at different magnifications. (Low: low magnification; High: high magnification).

The CSi scaffold was printed by SLA technology and had channels in both longitudinal and transverse directions (Figure 2A; Supplementary Figure S1). In order to adapt to the rat skull defect model, the scaffold was designed with a height of 2 mm and a diameter of 7 mm (Cooper et al., 2010). The channel diameter was 1 mm. This channel diameter can provide as much space as possible for new bone to grow into the scaffold. The longitudinal channel was uniformly distributed in a circular shape around the center of the scaffold, while the transverse channel run through the center of the scaffold. This three-dimensional channel structure effectively connected the surface and center of the scaffold, providing a pathway for cells migrating from all directions to the center of bone defects. The edge of the channel was not regular circles from overall surface morphology of the scaffold under SEM (Figure 2B). This is because calcium silicate undergoes shrinkage during the sintering process (Ding et al., 2022; Huang et al., 2023). Then we filled the CSH slurry and CuCl_2_ into the channel to obtain CSi/CS scaffold or CSi/CS/0.5Cu scaffold. Calcium sulfate was evenly loaded into the channel from the top view of three types of scaffolds (Supplementary Figure S2). And we scanned the cross-section of the CSi/CS/0.5Cu scaffold by SEM (Supplementary Figure S3). Calcium sulfate filled the channel and was combined with calcium silicate. There was no obvious gap on the joint surface of the two materials, which was attributed to the excellent adhesive property of calcium sulfate. We combined the three-dimensional channel structure with calcium sulfate to create a sandwich structure. The rapid degradation and bone conductivity of calcium sulfate will hopefully accelerate osteoblasts to enter the center of bone defect through three-dimensional channel structure (Yahav et al., 2020; Chen H. et al., 2022; Echave et al., 2022; Chopra et al., 2023). In addition, we carried out crystal phase detection on the scaffolds. As shown by the XRD pattern, the three groups had obvious diffraction peaks of wollastonite (PDF # 05–0586, PDF # 29–0376), indicating that the 3D-printed scaffold was mainly composed of calcium silicate (Figure 2C). In the CSi/CS group, the scaffold had a new peak of calcium sulfate dihydrate. In the CSi/CS/0.5Cu group, the diffraction peak of CuCl_2_ was added, which proved that CuCl_2_ was successfully loaded into the scaffold.

We also scanned the surface of the prepared three groups by electron microscopy, and obtained SEM results at different magnifications. Figure 2D showed that CSi maintained the calcium silicate crystal structure on the surface of the scaffolds after sintering, with a large number of pores and grooves. This finding was attributed to the fact that the photosensitive resin incorporated decomposed at high temperatures during the sintering process of ceramic blanks. Many irregular micropores were formed on the surface and inside of the scaffold. These micropores increased the porosity and roughness of the scaffold surface, which may contribute to the cell attachment (Calore et al., 2023; Ma et al., 2023). In contrast, the samples were immersed in calcium sulfate slurry and covered with a large amount of calcium sulfate with large crystal patterns for the CSi/CS group. These crystals were loosely connected and accompanied by a large number of small broken crystals, which may be one of the reasons for the rapid degradation of calcium sulfate. Finally, we observed that the surface of the CSi/CS/0.5Cu group no longer showed large crystals of calcium sulfate because of the addition of Cu^2+^, but a large number of dense small prismatic crystals instead. The surface of the scaffold was flatter than that of the CSi/CS group when viewed under low magnification. Next, we will study the changes in various biological functions of the 3D-printed calcium silicate artificial bone loaded with the calcium sulfate-Cu^2+^ delivery system in more detail.

### 3.2 Characterization of the scaffolds

**FIGURE 3:**
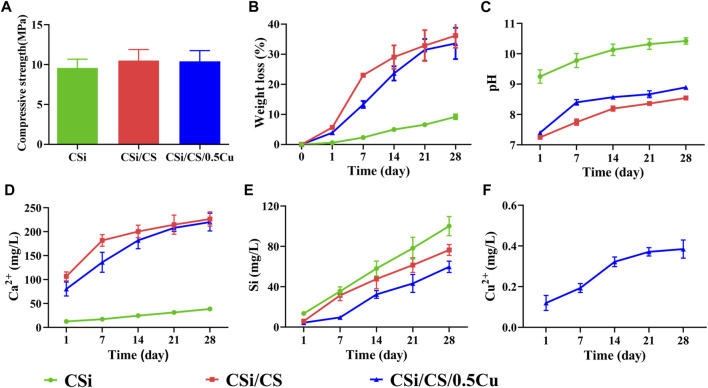
Physicochemical characterization of the scaffolds. **(A)** Compressive strengths. **(B)** The weight changes in scaffold degradation. **(C)** The pH of the scaffold changes in pure water. **(D)** Calcium ion release curve. **(E)** Silicon ion release curve. **(F)** Copper ion release curve.

For an ideal scaffold for bone repair, the scaffold should be mechanically strong enough to provide support for the defective area during bone repair. Our 3D-printed CSi had a compressive strength of up to 9.5 MPa (Figure 3A), which was sufficient to provide an initial structure and stability for the repair of cranial defects (Fu et al., 2013). Supplementary Supplementary Figure S4A showed the compression curves of three scaffolds. The compressive strength of the other two scaffolds was slightly stronger than that of the CSi group, but there was no significant difference. In addition, in terms of degradation performance, the weight loss of the CSi group after 4 weeks was 10% of the original weight. Moreover, the weight loss of the CSi/CS group and CSi/CS/0.5Cu group had values approximately 35%, which was due to the rapid degradation of calcium sulfate (Figure 3B). The data indicated that the CSi scaffold had good biodegradability compared to the conventional β-TCP scaffold (Jodati et al., 2020). We also measured the compressive strength of the three scaffolds after 4 weeks of degradation. Supplementary Figure S4B showed the compressive strength of the three scaffolds after degradation were 7.5 MPa (CSi), 7.8 MPa (CSi/CS), 7.9 MPa (CSi/CS/0.5Cu). None of the three scaffolds had an excessive decrease in compressive strength at 4 weeks of degradation. They can still provide certain mechanical support to the bone defect site.

The pH value of the surrounding environment will affect the behavior and growth of cells. Therefore, we also examined the change in pH value of the three groups (Figure 3C). The CSi group exhibited powerful alkaline properties at the beginning, notably reaching a pH of 10.4 after 4 weeks. This phenomenon can be detrimental to the adhesion and growth of cells on the material to some extent. However, the pH value of the CSi/CS group and the CSi/CS/0.5Cu group dropped below 9 after 4 weeks. This finding is explained by the introduction of calcium sulfate, which is able to effectively neutralize the alkalinity property of calcium silicate. This combination led to a significant decrease in pH.

In addition, we examined the concentration range of various ionic products in the scaffolds. The Ca^2+^ released from the CSi group at Day 28 was 38 mg/L, while the level of Ca^2+^ released from the CSi/CS group and CSi/CS/0.5Cu group was approximately 100 mg/L at Day 1 and rose to over 200 mg/L at Day 28 (Figure 3D). We clearly observed that the calcium silicate group had the lowest concentration of Ca^2+^ released due to the slower rate of calcium silicate degradation. In contrast, the release of Ca^2+^ in the CSi/CS group was the highest due to the rapid degradation of calcium silicate. The concentration of Ca^2+^ was slightly higher on the first day than in the cell culture media. The concentration continued to rise in the later period, but was still within the effective range for guiding bone regeneration (Chai et al., 2012). The calcium silicate degradation of the CSi/CS/0.5Cu group was slowed due to the addition of Cu^2+^, so the concentration of Ca^2+^ was slightly higher than that of the CSi group but lower than that of the CSi/CS group. Figure 3E shows the ionic concentration of silicon ions. The concentration of silicon released by the three groups of scaffolds was higher than that of normal plasma, but it could promote osteogenesis (Li et al., 2014). Finally, we also measured the release concentration of Cu^2+^ (Figure 3F), which was 0.38 mg/L on the 28th day. The Cu^2+^ concentration will not produce significant cytotoxic effects at such low release levels (Weng et al., 2017; Ameh and Sayes, 2019). This result demonstrates the slow-release effect of the calcium sulfate delivery system, which can stably release loaded drugs.

### 3.3 *In vitro* mineralization

**FIGURE 4:**
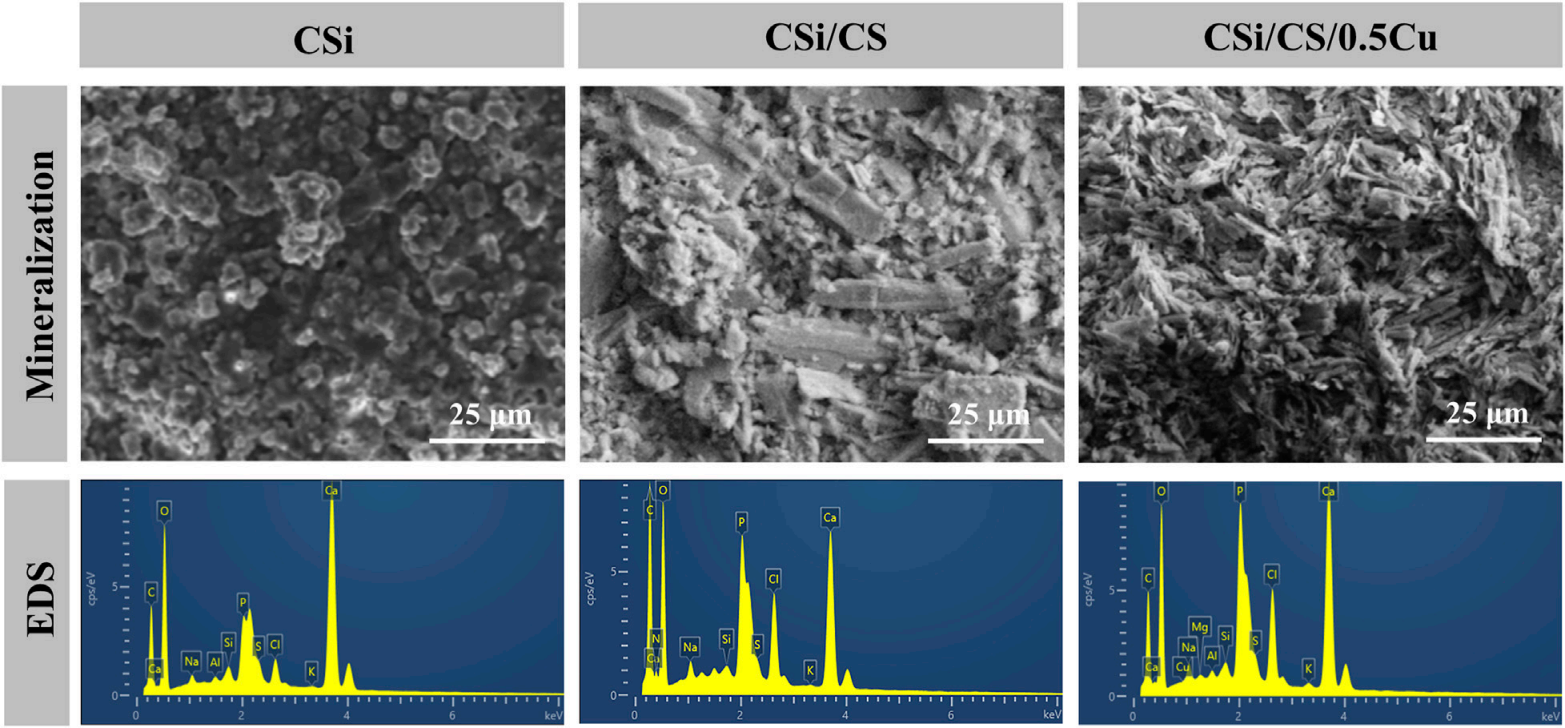
SEM images and EDS spectra of surface precipitates after soaking three groups in SBF for 1 week.

The formation of a hydroxyapatite layer is closely related to the osteogenic process, and the ability to form hydroxyapatite on the scaffold surface will directly affect the activity of osteoblasts (Xu et al., 2022). Therefore, we soaked the scaffold in SBF for 1 week and evaluated the ability of the scaffold to induce hydroxyapatite by an *in vitro* mineralization assay. Figure 4 shows the SEM images of the scaffold after soaking in SBF solution for 1 week. We found that the surface of the CSi group became less flat due to degradation and a small amount of hydroxyapatite was deposited. The CSi/CS group, on the other hand, clearly showed many agglomerates of tiny spherical particles distributed in the gullies. The CSi/CS/0.5Cu group also showed such agglomerates. To further verify whether the agglomerated particles formed on the surface were hydroxyapatite, we performed EDS scans on the scaffold surface. Based on the EDS spectra, the phosphorus content on the surface of the CSi/CS group and the CSi/CS/0.5Cu group was significantly higher than that of the CSi group. This finding indicates that loads of phosphorus were deposited on the surface of the scaffolds. Supplementary Table S3 summarized the results of EDS analysis, the amount of silicon, calcium, and phosphorus, as well as Ca/P of the deposited layer. The CSi group had a high Ca/P radio (9.1). In contrary, the CSi/CS group and the CSi/CS/0.5Cu group had a lower Ca/P radio (2.67, 2.71), which was closer to the Ca/P ratio (1.67, 2.71) of hydroxyapatite in the human body (Kien et al., 2018). This further proved that calcium sulfate can promote the generation of hydroxyapatite. This phenomenon is due to the degradation of calcium silicate to produce Si-OH groups that can become the nucleation center of hydroxyapatite. However, pure calcium silicate cannot rapidly expand the aggregation of hydroxyapatite. The dissolution of calcium sulfate produces a large amount of Ca^2+^, which accelerates the nucleation rate of hydroxyapatite (Huan and Chang, 2007). Thus, calcium sulfate can substantially enhance the biological activity of calcium silicate to strengthen the precipitation ability of hydroxyapatite on the surface. This combination may improve the osteogenic ability of calcium silicate *in vivo* (Wang et al., 2022; Liang et al., 2023).

### 3.4 The biocompatibility of the scaffolds with cells

**FIGURE 5:**
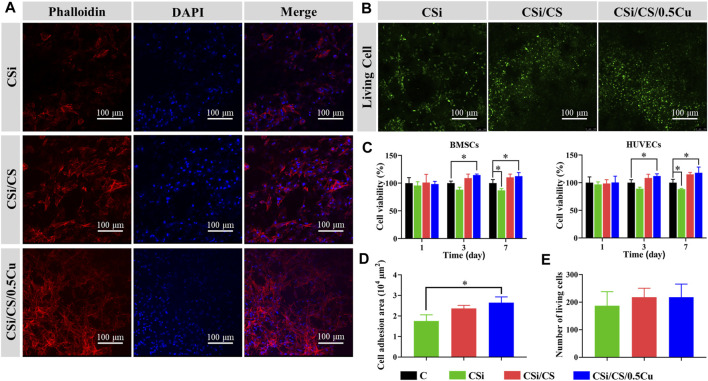
*In vitro* cytocompatibility of the scaffolds. **(A)** The adhesion of BMSCs to scaffolds was observed under confocal fluorescence microscope. Phalloidin staining of cell F-actin filaments was shown in red, and DAPI-stained cell nuclei was shown in blue. **(B)** Calcein AM staining showing the effect of scaffolds to the cell toxicity of BMSCs (green: live cells). **(C)** Cell activity of BMSCs and HUVECs were measured by MTT after 1, 3, and 7 days of culture with scaffold extract. Quantitative analysis of the cell area **(D)** and cell number **(E)** from the confocal fluorescence microscope imaging results. Data are presented as the mean ± SD (*n* = 3). (*, *p* < 0.05)

For a qualified biological scaffold, its biocompatibility is an important aspect that cannot be ignored. First, we assayed the ion concentrations in the conditioned media obtained from each scaffold. Supplementary Table S1, 2 show that the ion concentrations in the conditioned media were within the cell safe range (Chai et al., 2012; Li et al., 2014; Ameh and Sayes, 2019). Then, we treated the cells with conditioned media. On the third and seventh days, the MTT assay showed some promotion of cell proliferation in the CSi/CS group and the CSi/CS/0.5Cu group (Figure 5C). And both groups promoted the proliferation of cell by about 10% or more at day 7. This finding may be because the release of Ca^2+^ and Si ions from calcium silicate helped to enhance the viability and proliferation of BMSCs and HUVECs (Wang et al., 2019). In contrast, the CSi group showed the opposite effect and decreased by about 10% compared to the control group. The content of Ca^2+^ and Si ions in the conditioned medium of the three scaffolds was similar, but the conditioned medium of the CSi group was too alkaline. So we think that the alkaline nature of medium inhibited the proliferation of the CSi group (Aina et al., 2009). In the Supplementary Material, we also investigated the effect of CSi/CS/0.1Cu and CSi/CS/1Cu on cell viability. Supplementary Figure S5 shows that all treatments with Cu^2+^ positively affected cells after 3 days. However, cell viability showed a certain decrease in the CSi/CS/1Cu group compared to the CSi/CS/0.5Cu group. Therefore, we adopted CSi/CS/0.5Cu as the final scaffold composition in the experiment. To further confirm the effect of scaffolds on cell growth and adhesion, we also grew BMSCs on the surface of the scaffolds and stained the cytoskeleton of BMSCs with phalloidin to observe cell extension. In the confocal images (Figure 5A), we observed that the cells adhered to the surface of all three types of scaffolds, especially the BMSCs on the CSi/CS/0.5Cu scaffold showed good extension morphology similar to a starfish, and favorable cell activity. Afterward, we performed a quantitative analysis of the confocal images. We observed that the adhesion area of BMSCs on the CSi/CS/0.5Cu scaffold was improved compared to that of the CSi group (Figure 5D). This finding was also demonstrated by live cell staining experiments (Figure 5B), which indicated that cells survived normally on the surface of all three types of scaffolds. By counting the number of live cells in the field of view, we showed that there was no significant difference in the number of live cells on the three scaffolds (Figure 5E). These results proved that the CSi/CS/0.5Cu scaffold did not release toxic concentrations of ions in a short period to impair cell activity and that the degradation process of the scaffold did not negatively affect the normal growth of the cells.

### 3.5 Scaffolds promote osteogenic differentiation of BMSCs *in vitro*

**FIGURE 6:**
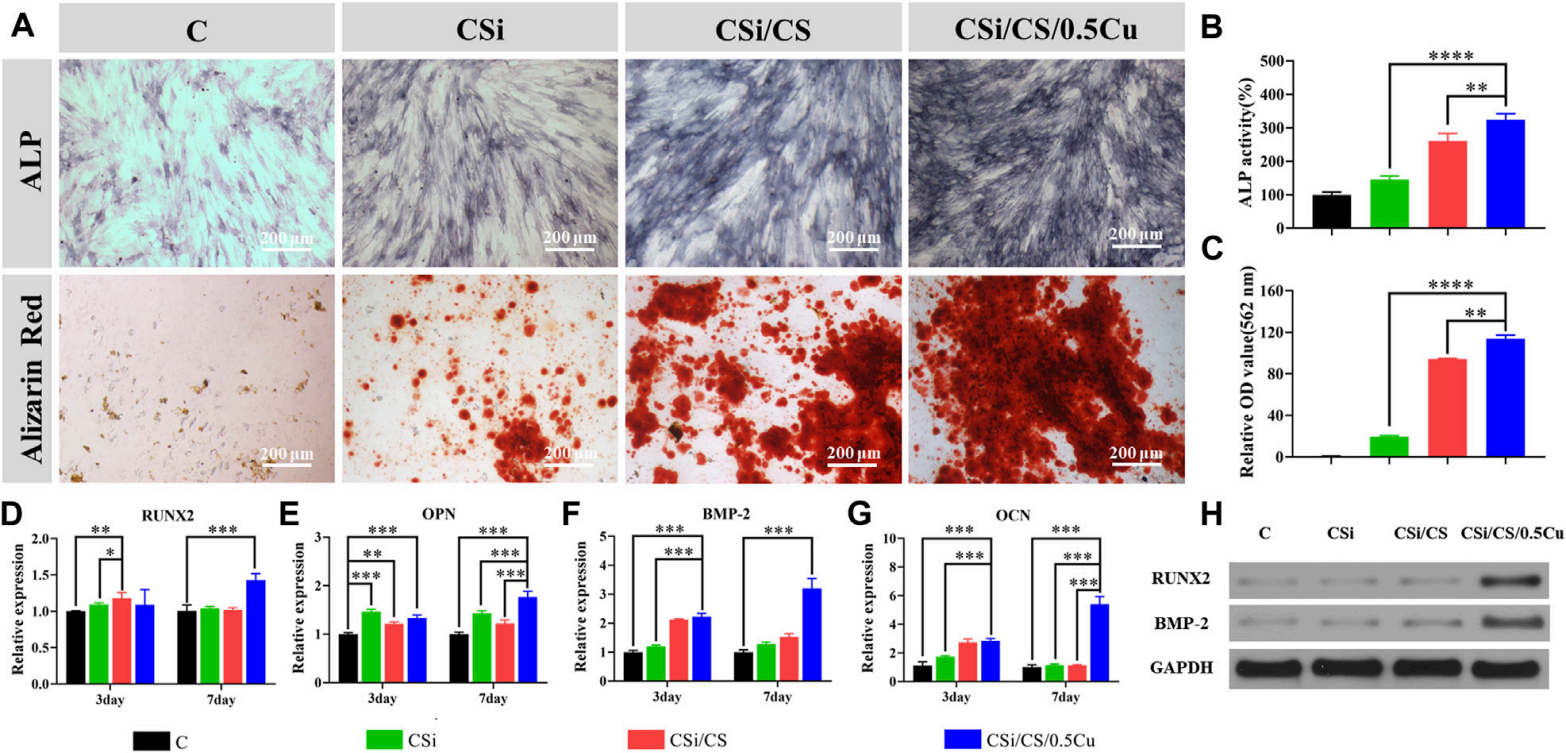
*In vitro* experiments on osteogenic differentiation of rat BMSCs. **(A)** The effects of different groups on osteogenesis differentiation of rat BMSCs in alizarin red staining and ALP staining. Quantitative analysis of ALP staining **(B)** and alizarin red staining **(C)**. RNA expressions of Runx2 **(D)**, OPN **(E)**, BMP-2 **(F)** and OCN **(G)** genes in BMSCs were detected by RT-qPCR after intervention with scaffold extracts. **(H)** BMSCs were cultured in conditioned media for the protein analysis of Runx2 and BMP-2. Data are presented as the mean ± SD (n = 3). (*, *p* < 0.05; **, *p* < 0.01; ***, *p* < 0.001; ****, *p* < 0.0001)

We next tested the ability of the 3D-printed calcium silicate artificial bone loaded with the calcium sulfate-Cu^2+^ delivery system to promote BMSC osteogenesis. First, we measured alkaline phosphatase activity to detect their early osteogenic differentiation status when BMSCs were cultured for 7 days (Vimalraj, 2020). According to the results (Figure 6A), the ALP staining in the CSi group and control groups was similar to that of the control group, with no significant differences. However, the ALP staining in the CSi/CS group and CSi/CS/0.5Cu group was significantly deepened, indicating a significant promotion of early osteogenic differentiation. Then, we used alizarin red staining as a marker of inorganic calcium to assess the efficiency of the mineralization phase (Maharjan et al., 2021). After BMSCs were cultured in the conditioned media for 14 days, a significant amount of mineralized nodule formation was observed by microscopy in the CSi/CS group and the CSi/CS/0.5Cu group. Then we quantitatively analyzed the results of ALP staining (Figure 6B) and alizarin red staining (Figure 6C). The ALP activity of the CSi group, CSi/CS group and CSi/CS/0.5Cu group was 145%, 260% and 323% higher than that of the control group. The OD values of alizarin red staining in the CSi/CS group and CSi/CS/0.5Cu group even reached about 100 times that of the control group. These results further confirmed that Cu^2+^ in concert with calcium sulfate can increase ALP activity and induce BMSC mineralization.

To further explain the effects of the CSi, CSi/CS, and CSi/CS/0.5Cu scaffolds on the osteogenic differentiation of BMSCs, we detected four osteogenic marker genes by RT‒qPCR (Figures 6D–G). Expression of the Runt-related transcription factor-2 (Runx2), osteopontin (OPN), bone morphogenetic protein-2 (BMP-2), and osteocalcin (OCN) genes is the key feature of osteoblasts at various stages of differentiation. These genes can be used to evaluate the functional status of osteoblasts under the intervention of 3D -printed calcium silicate artificial bone extracts. The relative expression of each gene at day 3 was Runx2 (1.096 ± 0.021, 1.181 ± 0.079 and 1.092 ± 0.209-fold), BMP-2 (1.203 ± 0.042, 2.121 ± 0.021 and 2.225 ± 0.113-fold), OCN (1.740 ± 0.069, 2.736 ± 0.247 and 2.855 ± 0.153-fold) and OPN (1.465 ± 0.046, 1.216 ± 0.035 and 1.328 ± 0.067-fold). Except for the expression of Runx2 gene, which was not a significant difference, the expression of the other three genes showed a distinct upward trend in the experimental group compared with the control group. As the duration of the experiment increased (7 days), Runx2 (1.041 ± 0.027, 1.022 ± 0.022 and 1.429 ± 0.089-fold), BMP-2(1.287 ± 0.059, 1.521 ± 0.113 and 3.196 ± 0.343-fold), OCN (1.149 ± 0.078, 1.139 ± 0.041 and 5.40 ± 0.543-fold) and OPN (1.432 ± 0.049, 1.220 ± 0.71 and 1.763 ± 0.122-fold) expressed higher in all experimental groups. Compared with the control group, the difference was statistically significant (*p* < 0.05). Runx2 is a critical gene in the process of osteogenic differentiation and initiates the differentiation process (Narayanan et al., 2019). The expression of Runx2 in the experimental groups was increased to a certain extent on the third day and the CSi/CS/0.5Cu group showed a significant upregulation of the Runx2 gene on approximately the seventh day, which may strongly promote osteogenic differentiation of BMSCs. OPN is a nonspecific early bone marker with an early peak during the proliferative phase and another peak after initial mineralization of the extracellular matrix, while BMP-2 and OCN are mainly associated with the later stages of osteogenic differentiation. The expression levels of these osteogenic differentiation-related genes change in response to the osteogenic differentiation of BMSCs and extracellular matrix formation (Salazar et al., 2016; Ching et al., 2017; Depalle et al., 2021). There was a higher trend of upregulation of BMP-2 and OCN gene expression in the experimental groups on the third day, while the gene expression in the CSi/CS/0.5Cu group had a highly significant increase on the seventh day. This phenomenon may be related to the elevated expression of Runx2 in the CSi/CS/0.5Cu group at the Day 7. Its upregulation as an upstream gene drove the elevated expression of BMP-2 and OCN genes one the seventh day, promoting the maturation of the extracellular matrix (Zhang et al., 2018). In order to further determine the changes in osteogenic related protein, we cultured BMSCs in conditioned media for 7 days and extracted cell proteins for the protein analysis of Runx2 and BMP-2. Both proteins were highly expressed in the CSi/CS/0.5Cu group compared to the control group (Figure 6H). There was also a slight increase in BMP-2 expression in the CSi/CS group at the Day 7.

All the results of BMSC differentiation demonstrated the potential of the CSi/CS/0.5Cu scaffold to promote bone regeneration. One of the explanations may be silicon. Many previous studies have reported that silicon stimulates osteoblast growth and contributes to osteogenesis by regulating osteogenesis-related genes (Götz et al., 2019; Srinath et al., 2020). Moreover, the Ca^2+^ concentration played a critical role in this process. This factor can regulate osteoblast differentiation by activating calcium-sensing receptors and increasing Ca^2+^ influx into osteoblasts (Chiu et al., 2023). In addition to these potential reasons, a recent study reported that Cu^2+^ could promote higher expression of osteogenesis-related genes, and mineralization of the cells (Burghardt et al., 2015). In terms of these aspects, our results were supported by previous studies, which further supported the ability of the CSi/CS/0.5Cu scaffold to enhance osteogenic differentiation.

### 3.6 Angiogenic capacity

**FIGURE 7:**
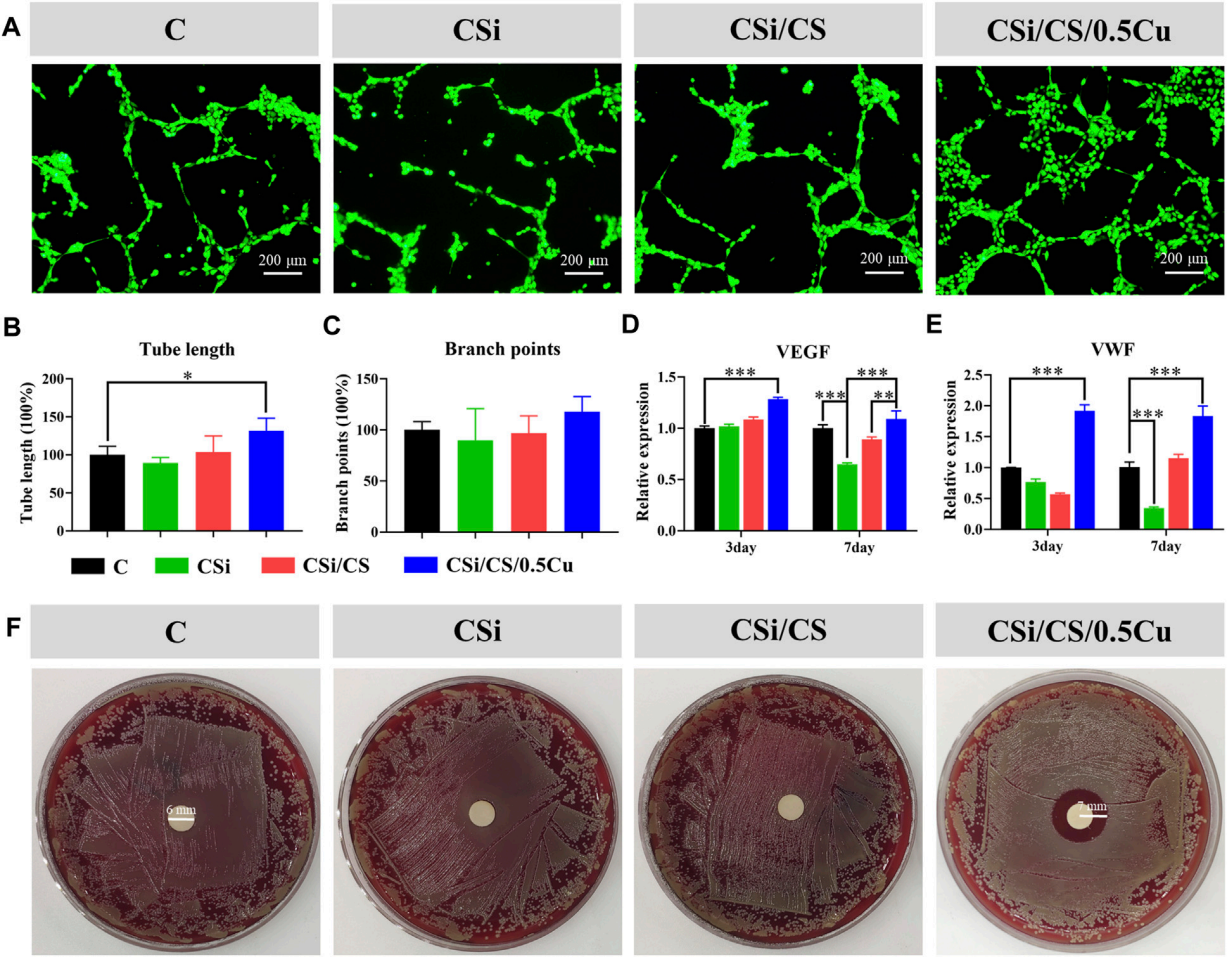
The pro-angiogenic and antibacterial effect of the scaffold extract. **(A)** Representative images of tube formation in different groups. Summarized data showing the difference of total length **(B)** and branch points **(C)** per field in HUVECs. Effects of scaffold extract on the expression of VEGF **(D)** and VWF **(E)**. **(F)** Antibacterial effect of the scaffold extract detected by disc diffusion method. Data are presented as the mean ± SD (n = 3). (*, *p* < 0.05; **, *p* < 0.01; ***, *p* < 0.001)

Angiogenesis which is an essential biological process, precedes osteogenesis during bone repair. This process is considered a prerequisite for bone regeneration. After measuring the osteogenesis of the 3D-printed calcium silicate artificial bone, we investigated its ability to support angiogenesis. The effect of scaffolds on the angiogenesis of HUVECs was assessed by the tube formation experiments. CSi group, CSi/CS group, and control group showed disorganization after 12 h, with HUVECs aggregating to form multiple strips but not complete tubules. In contrast, the CSi/CS/0.5Cu group was well-arranged and formed a meshwork of several small closed loops. The difference in the image results was noticeable (Figure 7A), demonstrating that the CSi/CS/0.5Cuscaffold had a significant ability to promote angiogenesis. The above conclusion was further confirmed by observing the total length of tubes and the number of nodes by ImageJ software analysis (Figure 7B, C). The total length of tubes formed in the CSi/CS/0.5Cu group was 1.3 times longer than that in the control group, and there was no significant difference between the other groups. The number of nodes formed by the tube also showed some improvement in the CSi/CS/0.5Cu groups, but no significant differences existed in the statistical analysis. As shown by the above results, the CSi and CSi/CS treatments had no effect on angiogenesis. In contrast, the CSi/CS/0.5Cu treatment could increase formation of the vascular networks by endothelial cells. This finding may be related to the fact that the CSi/CS/0.5Cu scaffold contains trace amounts of Cu^2+^. Cu^2+^ has been suggested in previous studies to promote angiogenesis through increased VEGF production. VEGF is a key signaling molecule regulating neovascularization, and its role as a critical indicator and regulator of angiogenesis has been well described (Das et al., 2022). The CSi/CS/0.5Cu scaffold slowly released free Cu^2+^ into the media, which may promote VEGF secretion, and induce HUVECs to form vascular networks. To further verify why the CSi/CS/0.5Cu scaffold promoted tubulogenesis, we examined the expression of VEGF and von Willebrand factor (VWF), which can promote angiogenesis (Figure 7D, E) (Carmeliet and Jain, 2011; Higgins et al., 2016). The relative expression of gene at day 3 was VEGF (1.017 ± 0.038, 1.086 ± 0.041 and 1.282 ± 0.034-fold) and VWF (0.765 ± 0.084, 0.569 ± 0.032 and 1.917 ± 0.172-fold). The relative expression of gene at day 7 was VEGF (0.648 ± 0.018, 0.889 ± 0.035 and 1.090 ± 0.112-fold) and VWF (0.345 ± 0.028, 1.152 ± 0.088 and 1.831 ± 0.229-fold). The results showed that the CSi/CS/0.5Cu group had upregulated expression of VEGF and VWF to produce proangiogenic synergistic stimulation and revascularization at each time point. In contrast, the CSi group showed some negative effects. This finding may be due to CSi significantly increasing the pH value of the media (Aina et al., 2009) The proliferation and cell function of HUVECs decreased under alkaline conditions for a long time. In summary, we demonstrated that the CSi/CS/0.5Cu scaffold upregulated VEGF and VWF and promoted the expression of VEGF in HUVECs, leading to a rapid angiogenic process *in vitro*.

### 3.7 Antibacterial ability

The inhibitory effect on *S. aureus* in the CSi group, CSi/CS group, and CSi/CS/0.5Cu group was evaluated by disc diffusion assays. As shown in Figure 7F, the CSi group and CSi/CS group did not show an observable inhibition zone. However, the inhibition zone of the CSi/CS/0.5Cu group was 4.0 mm, which was significantly different from that of the control group. The results indicated that the CSi/CS/0.5Cu scaffold had significant antibacterial activity against *S. aureus*. The antibacterial function of the CSi/CS/0.5Cu scaffold was supposed to be a result of Cu^2+^. This finding was consistent with the results reported in previous studies. Cu^2+^ can penetrate into bacteria, and the redox potential allows Cu^2+^ to generate hydroxyl radicals according to Haber-Weiss and Fenton reactions, which can lead to the destruction of lipids, proteins, and nucleic acids (Vincent et al., 2018). These results indicated that the CSi/CS/0.5Cu scaffold not only stimulates bone and angiogenesis but can also combat the infection from bone damage.

### 3.8 Evaluation of *in vivo* bone formation

**FIGURE 8:**
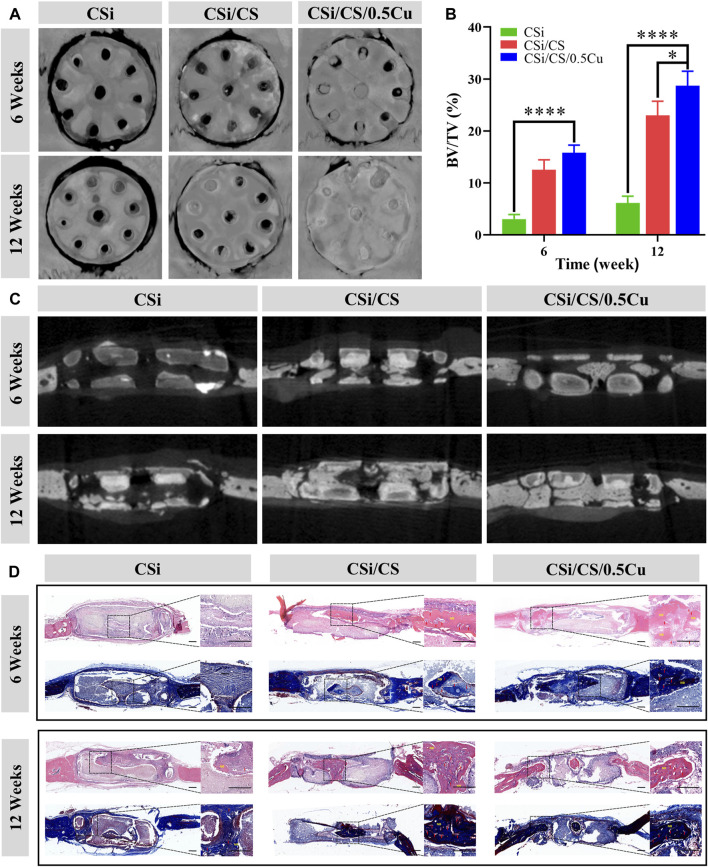
*In vivo* evaluation of scaffolds in a skull defect model. **(A)** 3D micro-CT images of the bone defect area. **(B)** Quantitative bone volume analysis based on micro-CT images. **(C)** Cross-section of micro-CT images of the bone defect area. **(D)** Representative images of H&E staining and Masson’s trichrome staining. Red arrow = blood vessel; NB = newly formed bone. Scale bars: 500 μm. Data are presented as the mean ± SD (n = 3). (*, *p* < 0.05; ****, *p* < 0.0001).

After the *in vitro* evaluation of the scaffold was completed in the above experiments, bone regeneration studies *in vivo* were conducted according to the planned protocol. The circular critical bone defect model was created on the rats’ skull with a ring drill. Throughout the experimental period, animals that underwent surgery survived with no signs of wound complications or infection. Animals were sacrificed at the 6th and 12th weeks, and the effect of bone repair *in vivo* was assessed by micro-CT and histological research. As shown in Figure 8A, C, representative 3D micro-CT images and 2D micro-CT images were obtained for all three groups of experiments. First, we clearly observed that the calcium sulfate had been wholly degraded at the sixth weeks, which did not affect new bone production. However, the calcium silicate was still not completely degraded at the 12th week, which can still provide sufficient mechanical and spatial support to the incompletely repaired bone defect area and accelerate the regeneration of the mineralized matrix during the bone repair process *in vivo*. Thus, this aspect is sufficient for the function of the 3D-printed calcium silicate artificial bone in mechanical support and osteogenesis induced by the tunnel. The bone repair results showed that the scaffolds generated more mineralized new bone in both the CSi/CS group and CSi/CS/0.5Cu group than the CSi group at the sixth week after implantation. The new bone generated on the scaffolds had filled the middle of the channels and had an initial connection with the edges of the bone defects. After 12 weeks of implantation, new bone tissue accumulated inside the scaffold channels. Especially in the CSi/CS group and CSi/CS/0.5Cu group, the channels were almost filled with new bone tissue. Notably, the new bone tissue of the CSi/CS/0.5Cu group was connected to the edges of the bone defect as a whole. This result suggested that direct osseointegration occurred between the scaffold and the natural bone. Next, we selected BV/TV as the indicator for quantitative analysis based on micro-CT images. As shown in Figure 8B, the proportion of new bone formation in the CSi/CS group and CSi/CS/0.5Cu group reached 12.5% and 15.8% respectively at sixth week. This indicator of both groups increased to 23% and 25.8% at 12th week. Both the CSi/CS group and the CSi/CS/0.5Cu group showed significantly higher BV/TV than the CSi group at both time points, which revealed significant osteogenic enhancement. The group with the best osteogenic effect was the CSi/CS/0.5Cu group, which was remarkably different from the CSi/CS group at the 12th week. This result further supported the previous findings.

To further evaluate the formation and mineralization of new bone during bone defect healing, we performed H&E staining and Masson’s trichrome staining on sections of cranial defects at the 6th and 12th weeks after implantation (Figure 8D). By analyzing the cross-sectional microphotographs of the bone defect sites at the 6th and 12th weeks, we found that both the CSi/CS group and the CSi/CS/0.5Cu group had increased new bone growth, and there was abundant vascularity in the middle of the new bone. Supplementary Figure S6 also demonstrates the angiogenic ability of the CSi/CS/0.5Cu group at the 12th weeks from *in vivo* experiments. The vascular distribution of the skull defect sites of the three groups was shown by vascular perfusion. The number of blood vessels in the CSi group, CSi/CS group, and CSi/CS/0.5Cu group increased significantly and sequentially. Especially in the CSi/CS/0.5Cu group, there were a large number of microvessels around the bone defect, which were due to the dual effect of silicon and Cu^2+^. In addition, we observed that the new bone formed in the channels of the CSi/CS/0.5Cu group was connected to the edges of the bone defect with good osseointegration. However, for the CSi group without calcium silicate filling, there was relatively little new bone formation at the defect site. Although the CSi group had a channel structure that created sufficient space for the implantation of new bone, the scaffold was still filled with inflammatory cells and fibrous tissue until the 12th week. There was less new bone production in channels. It is possible that the strong alkalinity of CSi scaffold can cause long-term inflammation or fibrous tissue to hinder the bone repair process (Maruyama et al., 2020; Newman et al., 2021). Therefore, we speculated that calcium silicate can reduce the alkalinity to reduce the inflammatory reaction in the CSi/CS group and CSi/CS/0.5Cu group or that an overly strong inflammatory response was subtly prevented from invading the scaffold due to the space being occupied by calcium silicate in the early repair process. Over time, calcium silicate was completely resorbed, and calcium sulfate still released silicon ions. The three-dimensional tunnel structure and silicon ions can recruit endogenous stem cells and endothelial cells from the body to the implant area. Then, vascularity and new bone production were induced in the channels. Thus, the rate of new bone formation was synchronized with the absorption of biomaterials. The addition of Cu^2+^ to the CSi/CS/0.5Cu group could also inhibit inflammation and promote osseointegration. Low concentrations of Cu^2+^ will be able to regulate the immune microenvironment of bone defect areas to decrease the inflammatory response (Lin et al., 2019). As shown in Supplementary Figure S7, CSi/CS/0.5Cu group extracts inhibited the expression of the inflammatory factor inductible nitric oxide synthase (INOS) and promoted the expression of anti-inflammatory related factor mannose receptor (CD206) after long-term culture with macrophages. This finding further validated that the CSi/CS/0.5Cu group can suppress inflammatory responses and promote osseointegration by modulating the immune microenvironment in the bone defect region. Taken together, these data demonstrated that the CSi/CS/0.5Cu group had a strong ability to promote angiogenesis and osteogenesis in large cranial defects.

## 4 Conclusion

In summary, this project designed a 3D-printed calcium silicate artificial bone improved by a calcium sulfate-Cu^2+^ delivery system (CSi/CS/0.5Cu). A calcium sulfate-Cu^2+^ delivery system would greatly enhance the osteogenic activity of calcium silicate to repair large bone defects. We successfully printed calcium silicate scaffolds with large sizes and precise structures using SLA technology. *In vitro* experiments showed that the calcium silicate artificial bone improved by the calcium sulfate-Cu^2+^ delivery system can not only provide excellent mechanical support and a cell growth environment for bone defect sites but also promote cell proliferation, stem cell osteogenic differentiation, angiogenesis and inhibit antibacterial growth. The results of the *in vivo* experiments were consistent with those of the *in vitro* experiments. We demonstrated that the improved calcium silicate artificial bone could induce blood vessels and osteoblasts into the center of large defects, and regulate bone regeneration at both times (different degradation rates of calcium silicate and calcium sulfate) and space (the three-dimensional tunnel structure). Thus, the new 3D-printed calcium silicate artificial bone improved by the calcium sulfate-Cu^2+^ delivery system provided a new strategy to study the promotion of bone defect repair and regeneration and can be applied in clinical bone repair engineering.

## Data Availability

The original contributions presented in the study are included in the article/Supplementary Material, further inquiries can be directed to the corresponding authors.
