# Supplementary material for: Calcium sulfate-Cu2+ delivery system improves 3D-Printed calcium silicate artificial bone to repair large bone defects
Source: Front Bioeng Biotechnol. 2023 Oct 25;11:1224557. doi: 10.3389/fbioe.2023.1224557 (PMC10634439; doi:10.3389/fbioe.2023.1224557)
Supplement: Supplementary file 1 [file DataSheet1.docx]

**Supplementary Material**

**Calcium Sulfate-Cu^2+^ Delivery System Improves 3D-Printed Calcium Silicate Artificial Bone to Repair Large Bone Defects**

Shijie Gao^1^, Jiawen Li^1^, Qingjian Lei^1^, Yan Chen^1^, Huayi Huang^1^, Feifei Yan^1^, Lingfei Xiao^1^, Tie Zhang ^2^, Linlong Wang *^1^, Renxiong Wei *^1^, Chao Hu *^1^,

1 Department of Spine Surgery and Musculoskeletal Tumor, Zhongnan Hospital of Wuhan University, 168 Donghu Street, Wuchang District, Wuhan, Hubei, 430071, China.

2 Wuhan QISIDA Technology Development Co., Ltd, Hi-Tech Park II Road 97, Wuhan, Hubei, 430223, China.

|  | Ca (mg/L) | Si (mg/L) | Cu (mg/L) |
| --- | --- | --- | --- |
| αMEM | 78.54±0.64 | 0.45±0.02 | 0 |
| CSi | 85.32±3.77 | 12.47±1.47 | 0 |
| CSi/CS | 197.87±6.63 | 7.53±0.51 | 0 |
| CSi/CS/0.5Cu | 176.43±4.88 | 5.64±0.94 | 0.15±0.02 |

**Table S1.** Ion concentration in αMEM control medium and αMEM conditioned medium of three groups.

|  | Ca (mg/L) | Si (mg/L) | Cu (mg/L) |
| --- | --- | --- | --- |
| ECM | 69.77±1.15 | 0.44±0.04 | 0.10±0.01 |
| CSi | 81.13±2.82 | 14.57±0.90 | 0.10±0.02 |
| CSi/CS | 218.63±6.50 | 7.37±0.31 | 0.11±0.01 |
| CSi/CS/0.5Cu | 195.20±5.75 | 4.87±0.29 | 0.22±0.02 |

**Table S2.** Ion concentration in ECM control medium and ECM conditioned medium of three groups.

|  | | Si (at.%) | | Ca (at.%) | P (at.%) | Ca/P (molar ratio) | |
| --- | --- | --- | --- | --- | --- | --- | --- |
| CSi | | 0.27±0.03 | 15.72±2.16 | 1.72±0.25 | 9.1 |  |  |
| CSi/CS | | 0.18±0.03 | 11.99±1.34 | 4.48±0.49 | 2.67 |  |  |
| CSi/CS/0.5Cu | | 0.17±0.02 | 13.30±2.35 | 4.91±0.37 | 2.71 |  |  |

**Table S3.** The percentage of each element and the molar Ca/P ratio of the deposited layer.


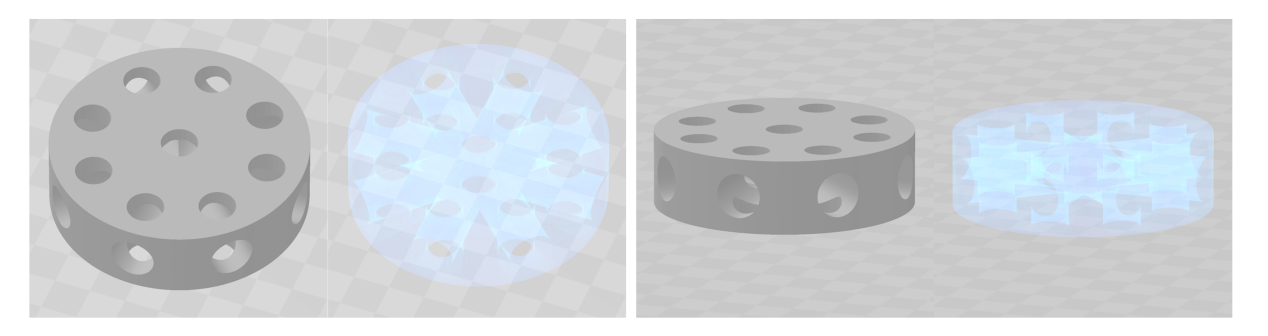


**Figure S1.** The 3D view and perspective view of scaffold. The cylinder scaffold is 2mm high and 7mm in diameter. The channel diameter is 1mm.


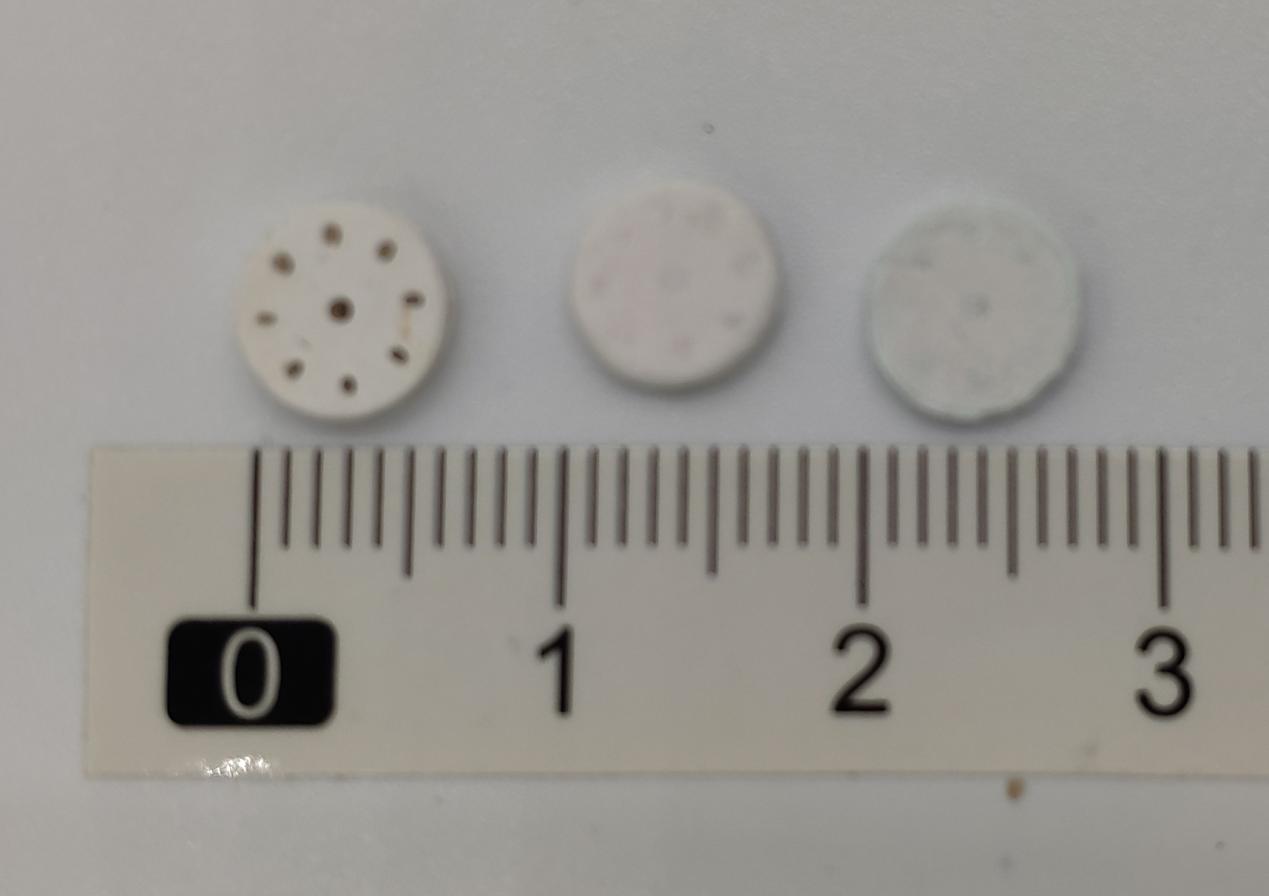


**Figure S2.** Top view of the 3D-printed scaffolds.


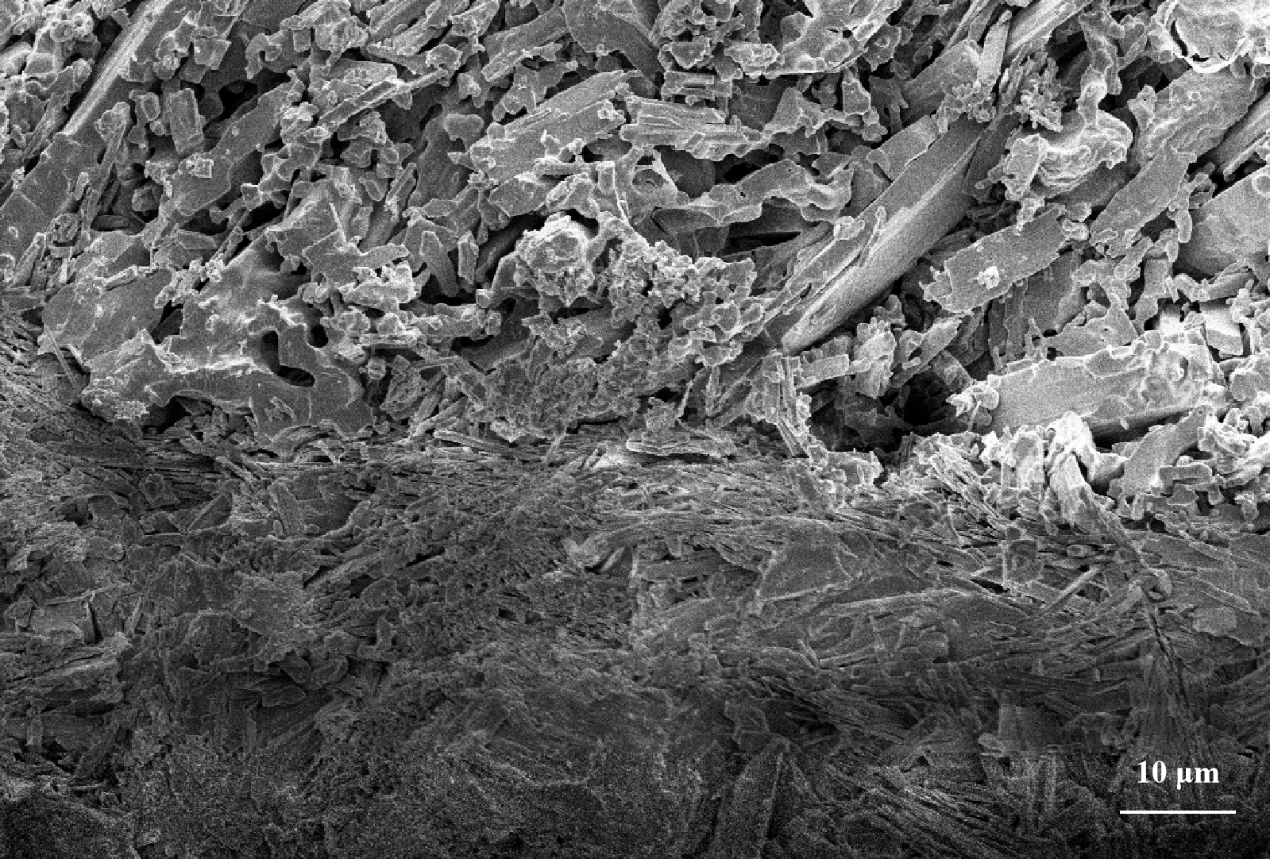


**Figure S3.** Cross-section at the junction of CSi scaffolds and CS/Cu


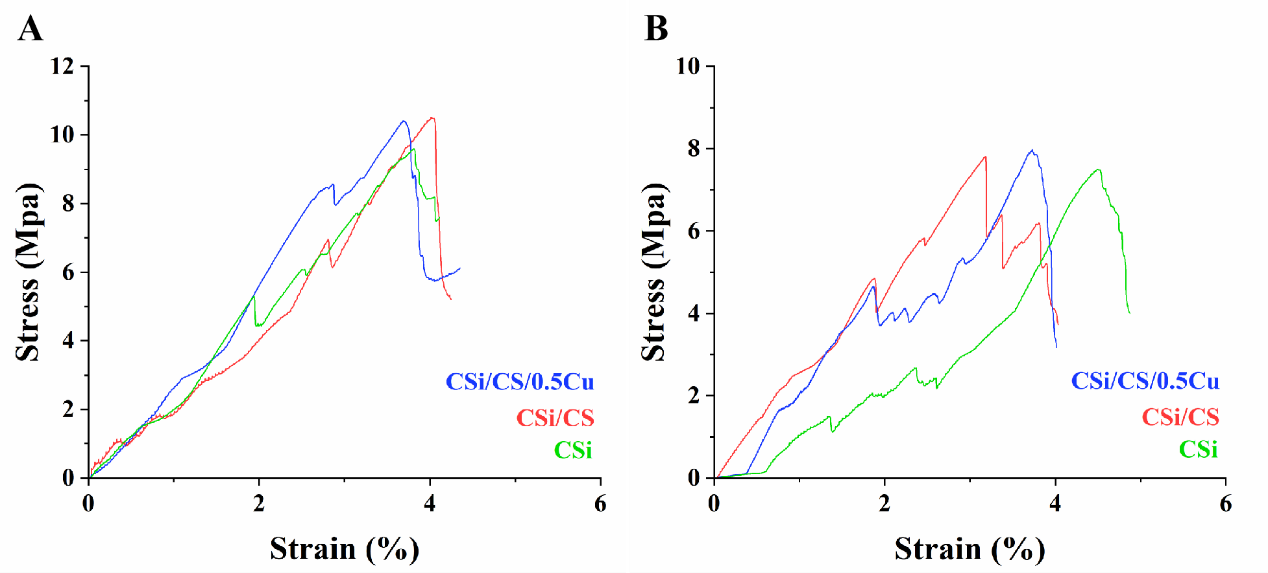


**Figure S4.** The compression curves: (A) Three types of untreated scaffolds. (B) Three types of scaffolds after 4 weeks of degradation.


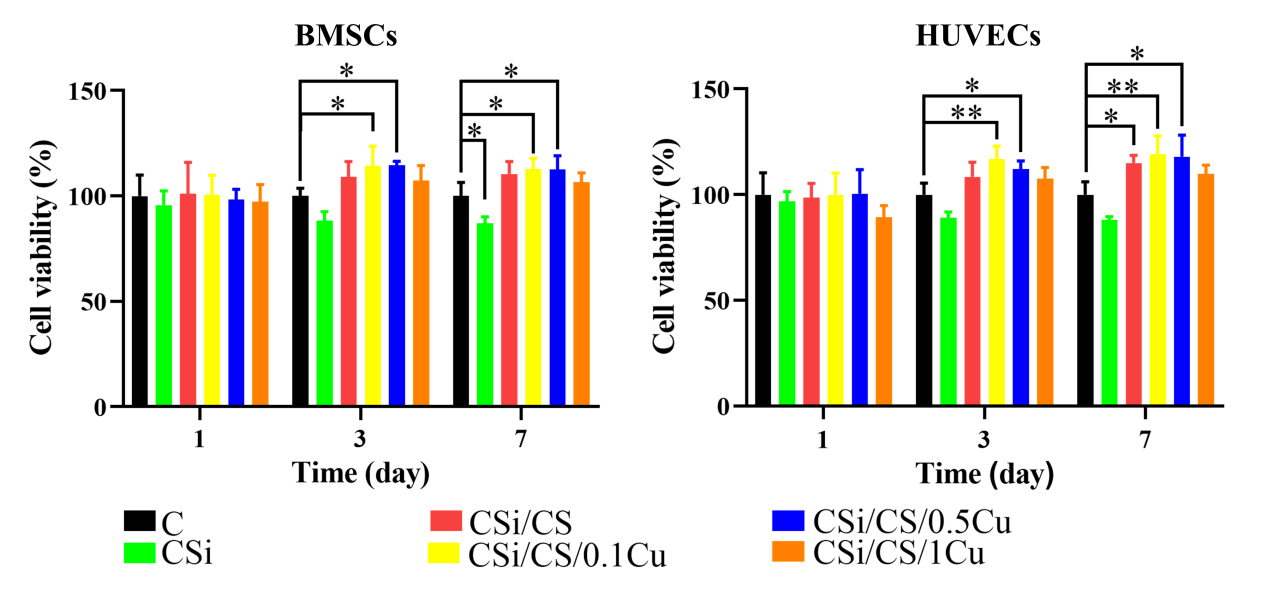


**Figure S5.** Cell activity of BMSCs and HUVECs were measured by MTT after 1, 3, and 7 days of culture with scaffold extract. Data are presented as the mean ± SD (n = 3). (*, p < 0.05; **, p < 0.01)


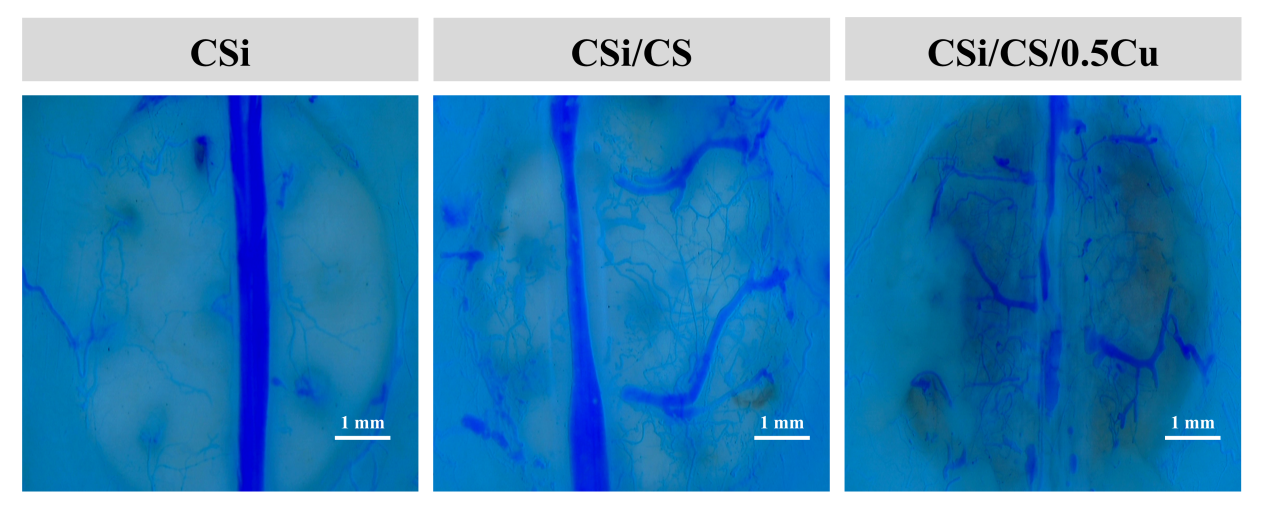


**Figure S6.** Visual observation of vascular growth on the surface of the bone defect after 12 weeks.


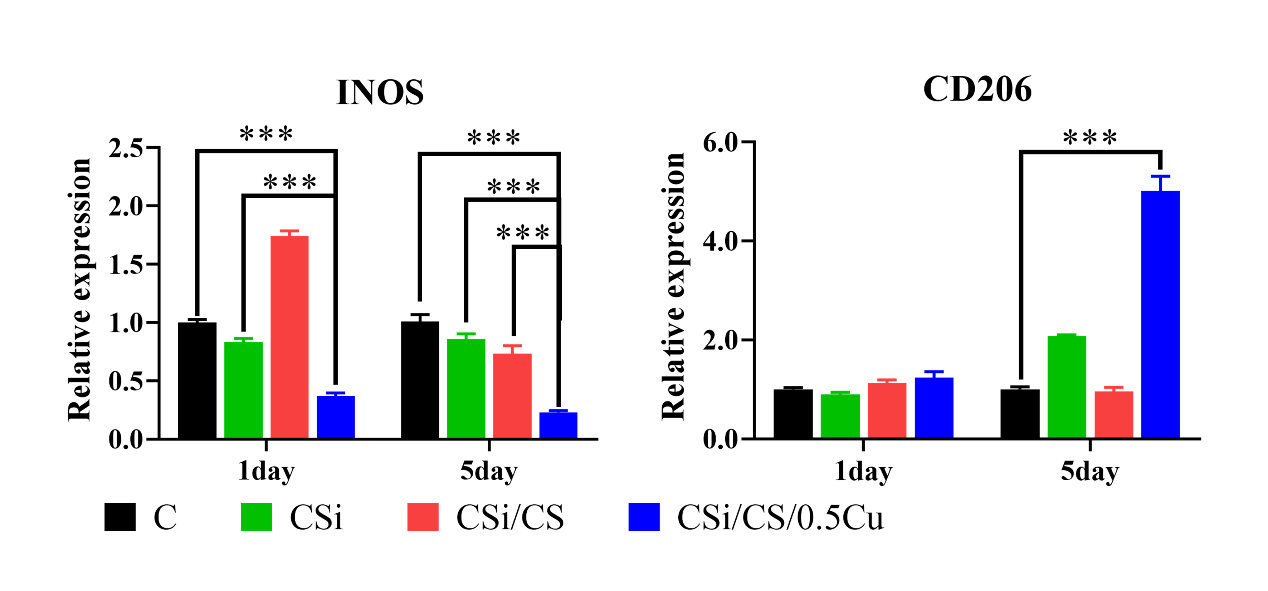


**Figure S7.** RNA expressions of Inflammation-related factors (INOS and CD206) in RAW 264.7 cells were detected by RT-qPCR after intervention with scaffold extracts.
